# Poly(*o*‑phenylenediamine) as an Organic Filler for Enhancing the Mechanical and Antibacterial Performance of Chitosan Films

**DOI:** 10.1021/acsabm.5c02312

**Published:** 2026-02-05

**Authors:** Mary Taylor, Jayla Jenkins, Mohammad Mohiuddin, Ufana Riaz

**Affiliations:** † Biomedical/Biotechnology Research Institute, 3066 North Carolina Central University, Durham, North Carolina 27707-3129, United States; ‡ Julius L. Chambers Biomedical/Biotechnology Research Institute (BBRI), 611247 North Carolina Central University, 1801 Fayetteville St., Durham, North Carolina 27707, United States; § School of Packaging, 115974 Michigan State University, 110 Packaging Building 448 Wilson Road, East Lansing, Michigan 48824-1223, United States

**Keywords:** chitosan, poly(*o*-phenylenediamine), DNA gyrase docking, disc diffusion, *B. subtilis*

## Abstract

Understanding structure–property relationships is essential for designing multifunctional biopolymer composites that integrate mechanical robustness, barrier performance, and antimicrobial activity in sustainable materials. Chitosan (CS) exhibits excessive hydrophilicity, limited mechanical strength, and poor moisture stability, which restrict its long-term performance in packaging applications. With the aim to enhance the mechanical strength, moisture absorption, and overall performance of CS, an organic aromatic polymer, poly­(*o*-phenylenediamine) (PoPD), was introduced into the matrix through in situ oxidative polymerization. Incorporation of PoPD improved the properties of CS by introducing aromaticity and electron delocalization, thereby limiting water uptake and molecular diffusion without relying on petroleum-derived additives. Remarkably, a low filler concentration (0.15 wt % of PoPD) produced drastic enhancement, in a tensile strength of 27.98 ± 1.40 MPa (as compared to 9.28 ± 0.46 MPa in neat CS) and an elongation at break value of 5.44 ± 0.27%. Moisture absorption studies confirmed a marked reduction at low filler levels, whereas higher PoPD contents generated compact morphologies that further restricted diffusion. Antibacterial evaluations revealed pronounced inhibition of *Bacillus subtilis* across all filler concentrations. Molecular docking analyses attributed this behavior to π–π-stacking, hydrogen bonding, and electrostatic interactions between PoPD and bacterial residues. The properties can be tuned by adjusting the filler content, producing multifunctional composites suitable for smart, sustainable packaging applications.

## Graphical abstract

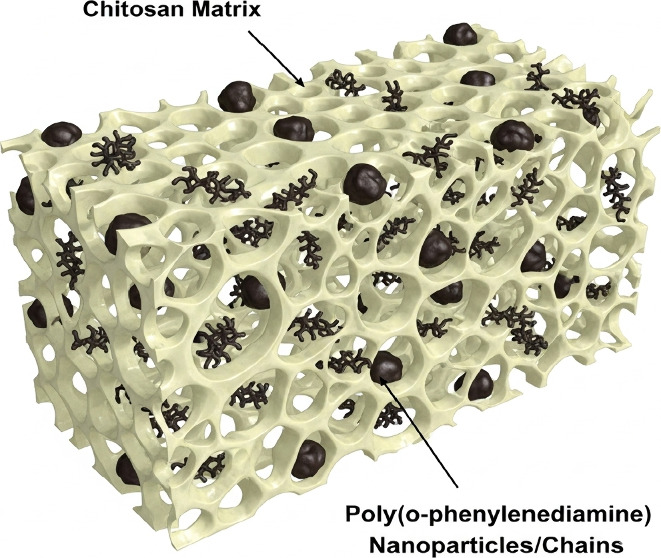

## 1 Introduction

The growing depletion of fossil fuel resources and the extensive dependence on petroleum-derived plastics for food packaging have been increasingly questioned in recent years.^1–3^ This challenge has intensified the search for sustainable, biodegradable materials with multifunctional capabilities that can contribute to food preservation. Chitosan (CS) is a biocompatible, naturally occurring polysaccharide predominantly derived from seafood shell waste and is widely regarded as a sustainable and renewable material. It is inexpensive, commercially available, and easily processed into coatings or films for food applications. However, CS-based films often exhibit poor mechanical strength and high moisture sensitivity, limiting their potential to replace conventional nonrenewable plastic packaging.^4–6^

Recent research has extensively explored the incorporation of organic fillers into chitosan (CS) films to enhance their mechanical strength, barrier properties, and functional performance for sustainable material applications.^7,8^ Conducting polymers such as polyaniline (PANI) and poly­(*o*-phenylenediamine) (PoPD) introduce aromaticity and electron delocalization, which strengthen interchain interactions and reduce hydrophilicity, leading to improved flexibility and barrier control.^9–14^ Mohammadi et al.^15^ synthesized PANI/CS films using molar concentrations of PANI ranging from 0.1 to 0.5 M concentrations. A maximum strength of 62.5 MPa was achieved at 0.5 M loading, compared to 34.5 MPa for pure CS. Thanpitcha et al*.* ^16^ prepared CS/PANI blends via solution casting to produce smooth, flexible films with robust mechanical properties at PANI loadings under 50 wt %. The conductivity was found to increase with higher PANI content, but HCl doping led to a 30% reduction in tensile strength to 39 MPa for the blend films, while the undoped blend exhibited a tensile strength value of 56.8 MPa. Anisimov et al.^17^ reported binary PANI–CS composites and ternary PANI/CS–poly­(vinyl alcohol) (PVA) composites, in which the PANI:CS ratios were varied (25:75, 50:50, 75:25) while keeping the PVA content constant. The Young’s modulus improved specifically as the CS fraction increased in the composite, indicating that CS served as the primary mechanical reinforcement for PANI-based materials. Vijayalekshmi and Khastgir^18^ investigated cross-linked CS/PANI–SiO_2_ hybrid membranes and showed that the tensile strength increased from 23 MPa (pure CS) to 35 MPa at 5 wt % PANI/SiO_2_, while elongation at break decreased from 42% to 28%. Collectively, these studies demonstrate that PANI, as an organic filler, significantly enhances the mechanical properties of pristine CS films, making them promising materials for packaging, biomedical devices, and electronic applications.

Despite the wide range of studies on chitosan-based composites with PANI, the incorporation of PoPD remains relatively unexplored.^13,14^ Importantly, most reports focus on higher filler loadings of PANI, which often result in particle agglomeration and brittle composites, limiting mechanical performance and homogeneity.^16,18^ This highlights the need to investigate low-level filler addition, which can potentially enhance mechanical, barrier, and functional properties without compromising film integrity or flexibility. The structure–property relationships governing the mechanical performance of PoPD/CS composite films also remain incompletely understood, motivating the present study.^11,13,14^ This work addresses a gap in the literature by demonstrating the effect of PoPD loading at low concentrations (0.5–1 wt %) on the spectral, mechanical, and antibacterial properties of the CS matrix. A series of CS/PoPD composite films were fabricated via in situ oxidative polymerization, with the objective of identifying PoPD concentrations that preserve homogeneity and maximize property enhancement. The structural and morphological features of the films were characterized using FTIR, UV–vis, fluorescence spectroscopy, XPS, and SEM, enabling the elucidation of the structure–property relationships between CS and PoPD.

## 2 Experimental Section

### 2.1 Materials

Chitosan (CS; medium molecular weight, 85% degree of deacetylation) was obtained from Fisher Scientific (USA). Acetic acid, *o*-phenylenediamine (OPD), ethanol, and distilled water were also purchased from Fisher Scientific (USA) and used without further purification.

### 2.2 Synthesis of PoPD/Chitosan

Chitosan (CS; medium molecular weight, degree of deacetylation ≈ 85%) was dissolved in 900 mL of 5 vol % aqueous acetic acid to obtain a polymer concentration of 13.6 g L^–1^. The solution was magnetically stirred at 95 °C for 24 h until a homogeneous, viscous solution was obtained. The apparent viscosity of the chitosan solution, measured at 25 °C by using a rotational viscometer at a shear rate of 10 s^–1^, was approximately 0.42 Pa·s. For composite film preparation, the aqueous OPD was dissolved in 5 mL of ethanol, while ferric chloride (FeCl_3_) was separately dissolved in 5 mL of ethanol at a 1:1 molar ratio relative to the aqueous OPD at ambient temperature (25 °C). In situ oxidative polymerization of poly­(*o*-phenylenediamine) (PoPD) was initiated by the simultaneous addition of OPD and FeCl_3_ solutions into 15 mL aliquots of the chitosan stock solution under continuous stirring. The PoPD loading varied at 0.15, 0.25, 0.50, 0.75, and 1.0 wt %, where wt % was relative to the dry mass of chitosan powder. After thorough mixing, the resulting dispersions were cast into leveled Petri dishes

### 2.3 Film Casting Procedure

Film casting was carried out under controlled laboratory conditions at a temperature of 25 ± 1 °C and a relative humidity of 50 ± 5%.^19^ For each sample, 15 mL of the prepared chitosan or PoPD/CS dispersion was cast onto a leveled Petri dish with an internal diameter of 90 mm, corresponding to a casting volume of approximately 2.4 mL cm^–2^. The dispersions were allowed to spread naturally without external shear to ensure uniform film formation. Drying was performed under ambient air circulation at the same temperature and humidity conditions for 48 h until constant mass was achieved. During the 48 h drying period at 25 ± 2 °C and 50 ± 5% relative humidity, ethanol was completely removed due to its high volatility. To eliminate excess ferric chloride and residual acetic acid, the dried films were gently rinsed with distilled water (three cycles, 25–30 mL per cycle) and subsequently air-dried under the same controlled conditions for an additional 24 h until constant mass was obtained. Following drying, the films were carefully peeled from the casting substrate. Prior to mechanical and moisture-related testing, all films were conditioned at 25 °C and 50% relative humidity for 48 h to equilibrate moisture content and ensure comparability of property measurements across samples.

## 3 Characterization

### 3.1 Colorimetric Properties of Films

The colorimetric properties of the films were evaluated using the CIELAB color space, with L* representing lightness, a* the red–green coordinate, and b* the yellow–blue coordinate.^20^ Digital images of the films were analyzed to extract these values at the center of each sample, and the total color difference (ΔE* – *ab*) was calculated relative to the reference film (Film CS) using the CIE76 equation:

$$\Delta E_{ab}^{*}=\sqrt{(L_{2}^{*}-L_{1}^{*}{)}^{2}+(a_{2}^{*}-a_{1}^{*}{)}^{2}+(b_{2}^{*}-b_{1}^{*}{)}^{2}}$$

The reported values represent the average of multiple measurements to account for any local heterogeneity across the film surface.^20^

### 3.2 Fourier-Transform Infrared (FTIR) Spectroscopy

Fourier-transform infrared (FTIR) spectra were collected using a PerkinElmer Spectrum spectrometer equipped with a diamond ATR crystal over the wavenumber range of 4000–500 cm^–1^. Each spectrum was acquired by averaging 16 scans at a spectral resolution of 4 cm^–1^. The resulting spectral data were processed and analyzed using Origin-Pro software (Origin Lab Corporation, Version 2024).

### 3.3 Ultraviolet Visible and Fluorescence Spectroscopy

UV–vis spectroscopy was collected on a Shimadzu UV–visible spectrometer (Kyoto, Japan). Fluorescence emission spectra were recorded in the wavelength range of 280 nm–550 nm on a fluorescence spectrophotometer model Fluorolog3, Horiba Scientific, Irvine, CA, USA. Spectral data were analyzed using Origin Lab Corporation’s OrginPro (Version 2024). The optical bandgap of the films was estimated from the UV–vis absorption spectra using the Tauc method.^21^

### 3.4 X-ray Photoelectron Spectroscopy

XPS experiments were performed using a Physical Electronics Versa Probe III instrument equipped with a monochromatic Al kα X-ray source (*h*ν = 1,486.6 eV) and a concentric hemispherical analyzer.^22^ Charge neutralization was performed using both low-energy electrons (<5 eV) and Ar ions. The binding energy axis was calibrated using sputter-cleaned Cu (Cu 2p_3/2_ = 932.62 eV, Cu 3p_3/2_ = 75.1 eV) and Au foils (Au 4f_7/2_ = 83.96 eV). Peaks were charge-referenced to the CH_*x*_ band in the carbon 1s spectra at 284.8 eV. Measurements were made at a takeoff angle of 45° with respect to the sample surface plane. This resulted in a typical sampling depth of 3–6 nm (95% of the signal originated from this depth or shallower). Quantification was done using instrumental relative sensitivity factors (RSFs) that account for the X-ray cross section and inelastic mean free path of the electrons. On homogeneous samples, major elements (>5 atom %) tend to have standard deviations of <3%, while minor elements can be significantly higher. The analysis size was ∼200 μm in diameter.

### 3.5 Scanning Electron Microscopy (SEM)

The cross-sectional microstructures and top surfaces of the film samples were investigated using an FEI XL30 SEM-FEG scanning electron microscope (FEI, Hillsboro,USA).

### 3.6 Dynamic Mechanical Analysis (DMA)

DMA was preformed using a TA Instruments DMA Q800. Rectangular specimens (10 × 10 mm^2^) were cut from the cast films by using a sharp blade to minimize edge defects. The thickness of each specimen was measured at five different points using a digital micrometer, and the average thickness was recorded. Prior to testing, all specimens were conditioned at 25 ± 2 °C and 50 ± 5% relative humidity for 48 h to equilibrate moisture content and ensure reproducibility. Tensile testing was performed using a universal testing machine in accordance with ASTM D882 at a crosshead speed of 5 mm min^–1^. Each measurement was repeated in triplicate, and the average values of tensile strength, elongation at break, and Young’s modulus were reported. Mechanical stress was applied at a controlled rate of 1 N/min, with measurements taken until the maximum load capacity of 18 N was reached.

### 3.7 Tensile Testing analysis

Tensile properties of the films, including tensile strength, elongation at break, and Young’s modulus, were measured by an external facility (Materials Characterization Laboratory, Penn State University) using an MTS Criterion universal testing machine equipped with a 50kN load frame. The tests were conducted in accordance with ASTM D882.

### 3.8 Moisture Absorption Studies

Moisture absorption studies were carried out following standard procedures commonly reported in the literature.^23^ The films were first cut into uniform specimens (1–2 cm^2^), and their thickness was measured using a digital micrometer. Prior to testing, each sample was thoroughly dried in a vacuum oven or a desiccator containing a drying agent such as DRIERITE for 24–48 h until a constant dry weight (*W*_0_) was obtained. After drying, the samples were placed in a controlled humidity chamber prepared using a saturated NaCl solution to achieve a fixed relative humidity (typically 75% RH). At predetermined time intervals, the samples were removed, gently blot-dried to eliminate only surface water, and immediately weighed to obtain the moisture-absorbed weight (*W*_t_). The percentage of moisture absorption was then calculated by using (*W*_t_ – *W*_0_)/*W*_0_ × 100. For each composition, a minimum of three to five replicates were tested, and the results were expressed as mean ± standard deviation, with statistical comparisons performed using one-way ANOVA.

### 3.9 Antioxidant Studies

Free radical generation was tested using a 1,1-diphenyl2-picryl hydrazyl (DPPH) technique. A total of 2 mg of DPPH was dissolved in 1 L of methanol for making the stock solution. Filtration of the DPPH stock solution using methanol yielded a usable mixture with an absorbance of around 0.973 at 517 nm. In a test tube, 2 mL of DPPH workable solution was combined with 10 μL of PoPD/CS solution in water. Ascorbic acid was used as a positive control under identical experimental conditions to validate the antioxidant assay. The reaction mixtures were incubated in complete darkness for 30 min, after which the absorbance was measured at 517 nm. The following formula was used to compute the percentage of antioxidants: % of antioxidant activity= [(*A*_c_ – *A*_s_) ÷*A*_c_] × 100, where *A*_c_ is the control reaction absorbance, and *A*_s_ is the testing specimen absorbance. A calibration curve based on ascorbic acid concentration was used to confirm assay linearity (Supporting Information Figure S1). A linear plot of % inhibition versus concentration was analyzed using the equation of a straight line: *y* = *mx* + *c*, where *x* is the concentration of the measured substance, and *y* is the % inhibition.

### 3.10 Antimicrobial Studies

Antimicrobial activity was evaluated against *Bacillus subtilis*, a well-established Gram-positive model organism commonly used in food-packaging-related antimicrobial studies due to its well-characterized cell wall structure and relevance for assessing chitosan-based materials. The antimicrobial activities of the samples were studied using a disc diffusion technique, where inhibition zones were observed. To make the composite into discs, a 5 mm diameter stainless steel puncher was pressed into films. Inhibition zone tests were performed on Gram-positive *Bacillus subtilis* cells. Briefly, freshly grown *B. subtilis* cells with an optical density (OD at 600 nm) of ∼0.55 were washed with PBS twice, resuspended, and further diluted in PBS to an approximate OD_600_ 0.016. Aliquots of 150 μL of the diluted cells were spread on LB agar plates and kept in a biological safety hood for 5–10 min before placing the composite film discs evenly spaced on agar plates. The plates were then incubated at 37 °C for 24 h. The diameter of the inhibition zones was measured in centimeters (cm) across the clear zone through the center of each disc using a sterile ruler. The percentage zone of inhibition (% ZOI) was calculated using the equation: % ZOI = (average inhibition zone diameter/maximum inhibition zone diameter) × 100. Statistical evaluation of zone diameters was carried out using one-way ANOVA followed by an appropriate posthoc test (e.g., Tukey or *t-*test), performed using Origin-Pro 2024. Results were reported as mean ± standard deviation. A two-sided significance level of *p* < 0.05 was used for assessing statistical significance.

### 3.11 Docking Studies

Molecular docking studies were conducted to validate the binding region of the Bacillus subtilis protein (PDB ID: 4DDQ) with the ligands. The protein structure was retrieved from the Protein Data Bank (http://www.pdb.org) and prepared by the addition of polar hydrogens and assignment of Kollman united-atom charges.^22^ Ligand structures—monomeric OPD, chitosan (CS), and the PoPD/CS composite—were energy-minimized and assigned Gasteiger partial charges before being saved in PDBQT format. To represent the polymer, a short oligomeric fragment (3–5 repeating units) of PoPD–CS was used, which is a standard approximation for docking studies involving macromolecular ligands. Docking simulations were performed by using AutoDock Vina version 1.2.3. A 3 × 3 × 3 Å^3^ grid box was generated with AutoGrid and centered on the active binding region of the *B. subtilis* 4DDQ protein to allow full rotational flexibility of each ligand. For each ligand, 10 binding modes (poses) were computed. The docked complexes were ranked based on binding affinity, and the conformation with the lowest binding energy was selected for detailed interaction analysis, including hydrogen-bonding patterns and hydrophobic interactions within the active pocket.

## 4 Results and Discussion

When OPD is oxidized by FeCl_3_, it forms radical cations, as shown in Scheme 1b. The positive charge on the radical cation is delocalized across the aromatic ring, and repetition of this step-growth process yields an amine-rich OPD backbone, as shown in Scheme 1b. The PoPD chains associate with CS through network formation between PoPD (filler) and CS (matrix) composite films, which are depicted schematically in Scheme 1c.^24^

**Scheme 1:**
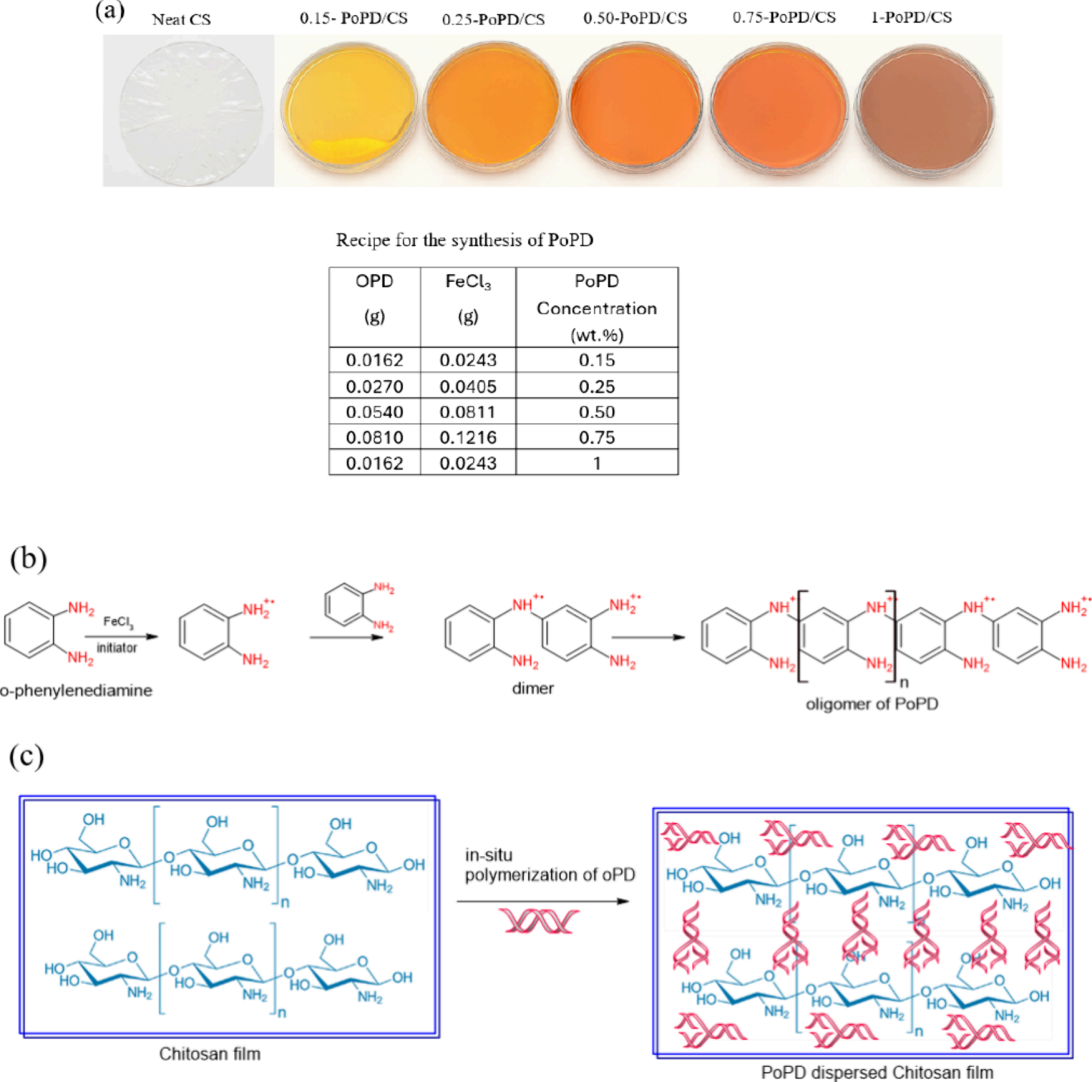
(a) Synthesis of PoPD/CS Composite Films, (b) Mechanism of Polymerization of OPD, and (c) Schematic Depiction of Formation of PoPD Reinforced CS Composite Films

Colorimetric determination of the films showed a decreasing trend in lightness from the CS film (L* = 82.55) to the 1-PoPD/CS composite film (L* = 58.55), indicating that the films become progressively darker with increasing filler concentration or thickness (given in Supporting Information in Table S1). The a* values increased from the CS film to the 0.5-PoPD/CS composite film, reflecting a shift from a neutral yellow toward a more reddish-orange hue. The b* values, representing yellowness, peaked at lower filler content and then declined as the films developed darker, more saturated brown/umber tones. The overall color change from pristine Cs to the 1-PoPD/CS composite film was 51.73, representing a very large perceptual difference (Δ*E* > 5 detectable by the human eye). The most pronounced change occurred between the first and second films, indicating that even small additions of filler produce noticeable color variation.^25^

### 4.1 Confirmation of Composite Structure Using IR and XPS Analysis

FT-IR analysis was employed to verify the chemical structure of the PoPD/CS composite films (given in Supporting Information in Figure S2). The IR spectrum of neat CS showed a broad peak at 3349 cm^–1^ attributed to O–H stretching and N–H stretching vibrations of the polysaccharide moieties, while the peak at 2927 cm^–1^ was due to aliphatic C–H bond stretching vibrations. The distinct peaks near 1640 and 1590 cm^–1^ corresponded to amide I (C=O stretching) and N–H bending of free amino groups, respectively. Upon incorporation of PoPD, additional peaks appeared in the 1500–1630 cm^–1^ range, characteristic of C=C stretching in aromatic rings. The peak at 1250 cm^–1^ was associated with the C–N stretching vibration of aromatic amine. The observed attenuation and slight red shift in the broad O–H/N–H stretching band suggested the formation of hydrogen bonding/covalent interactions between PoPD and CS.^22^ These spectral modifications collectively indicate that polymerization of OPD occurs in close association with the CS framework, leading to a well-integrated hybrid network that confirms successful composite formation and strong interphase compatibility.

XPS analysis was conducted to explore the interaction between the functional groups of CS and PoPD. High-resolution spectra of C 1s, O 1s, and N 1s regions were deconvoluted for each element to identify their subpeak components. Literature reports that the C 1s spectral line of neat CS typically has three components: −C–H (285.0 ± 0.2 eV); C–C (286.4 ± 0.2 eV); and C–O–O-H (288.4 ± 0.2 eV).^26^ The C 1s XPS spectra of the composite films containing 0.15, 0.25, 0.50, 0.75, and 1 wt % PoPD showed overlapping peaks that coincided with reported values.^26^ As shown in Figure 1(a-e), the C 1s spectra of the composite films were deconvoluted into C–H (284.8 eV ± 0.2 eV), C–(O/N) (285.7 ± 0.2 eV), C–O (286.7 ± 0.2 eV), and COO (288.7 ± 0.2 eV). The low-binding-energy C–H component represented aromatic C from PoPD and aliphatic carbons from CS backbones, while the C–(O/N) components represented C atoms singly bonded to O/N, confirming interactions with OH/NH_2_ functionalities. The higher-energy C–O and COO peaks were attributed to C=O and COO species. With increasing OPD concentrations (0.15 → 1 wt %) the C–H peaks intensified, whereas the C–(O,N) and C–O peaks diminished, indicating a shift from an O-rich CS surface to one dominated by the aromatic, conjugated PoPD backbone. The N 1s spectra in Figure 1f–j displayed two distinct peaks corresponding to N–C (399.6 eV ± 0.2 eV) and N–C^+^ (401.2 ± 0.2 eV), representing a neutral and protonated nitrogen. As the PoPD content increased, the relative intensity of the N–C^+^ peak increased, depicting enhanced protonation and doping of the polymer chains.^22^ The population of positively charged N sites reflected stronger delocalization within the PoPD network, supporting improved charge transport in the films. The O 1s spectra in Figure 1k–o were resolved into three major peaks at 531.6 eV ± 0.2 eV (C–O), 532.8 eV ± 0.2 eV (OCO), and 533.8 eV ± 0.2 eV (−OH/H_2_O). The progressive decrease in the C–O components with increasing OPD concentrations suggested reduced OH and C–O–C contributions from the CS phase.^22^ Overall, the results indicate that higher PoPD loading leads to a surface that is more electronically active, with PoPD predominantly localized at the film surface.

**Figure 1:**
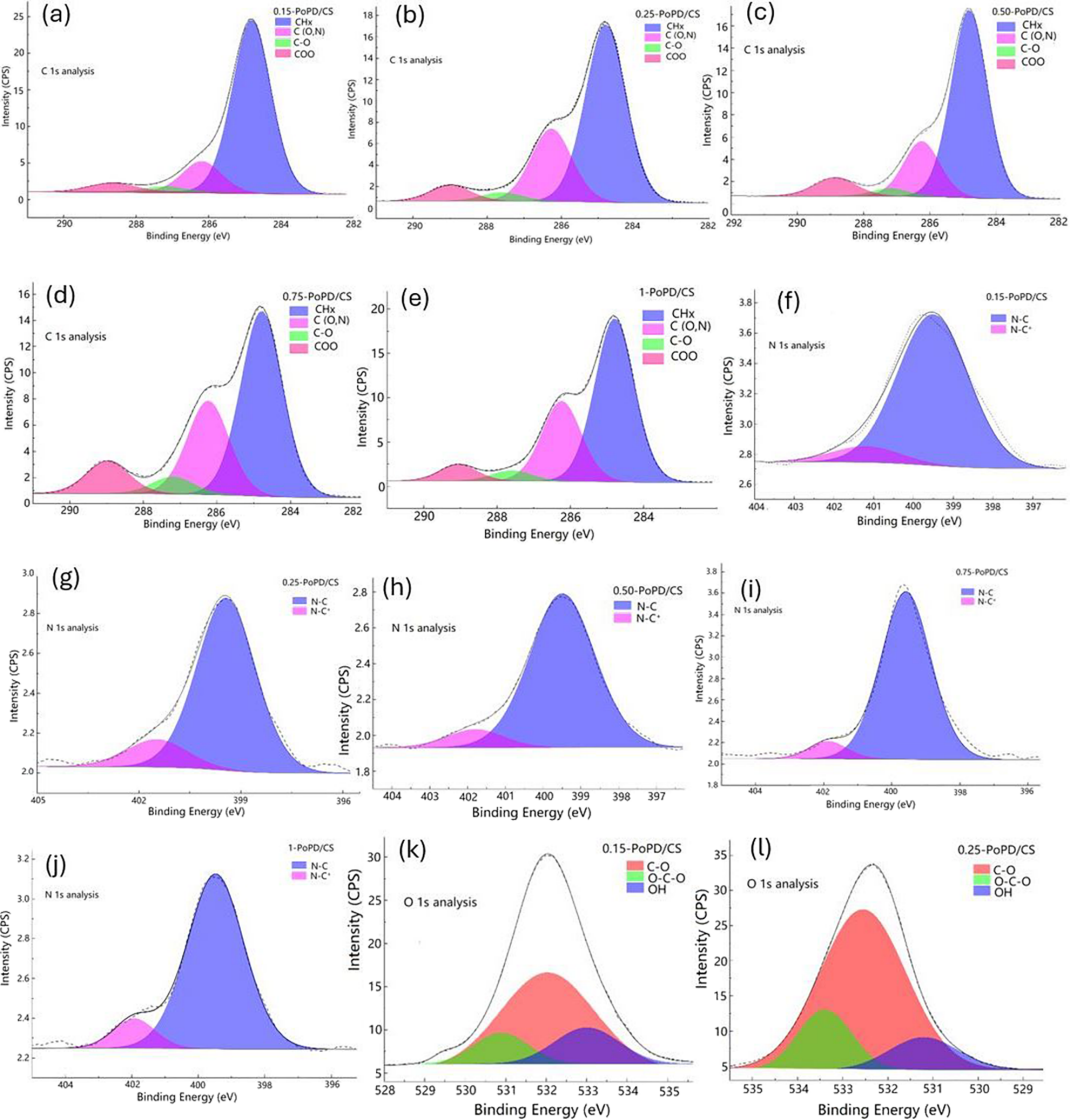
XPS of C 1s for (a) 0.15-PoPD/CS, (b) 0.25-PoPD/CS, (c) 0.5-PoPD/CS, (d) 0.75-PoPD/CS, and (e) 1-PoPD/CS; N 1s for (f) 0.15-PoPD/CS, (g) 0.25-PoPD/CS, (h) 0.5-PoPD/CS, (i) 0.75-PoPD/CS, and (j) 1-PoPD/CS; and O 1s for (k) 0.15-PoPD/CS, (l) 0.25-PoPD/CS, (m) 0.5-PoPD/CS, (n) 0.75-PoPD/CS, and (o) 1-PoPD/CS.

### 4.2 UV–Vis and Bandgap Studies

Figure 2 presents the UV–visible absorption spectra of the PoPD/CS composite films. The UV–vis absorption spectra exhibited a strong peak at 329 nm in the high-energy ultraviolet region, which is primarily associated with π → π* electronic transitions of the C=O moieties and the N-acetylglucosamine units within its molecular structure,^18^ as shown in Figure 2. The addition of PoPD showed increased wavelength absorption and a shift in the peak region, indicating extended conjugation from the aromatic PoPD. For PoPD/CS composite films, a second, broader absorption band typically appeared in the visible region, around 400–480 nm, and was attributed to n → π* electronic transitions arising from C=N or charged quinonoid and phenazine-like structures generated during the polymerization process.^22,24,27^ This feature was also indicative of charge-transfer exciton-type transitions along the conjugated polymer backbone, reflecting extended delocalization of electrons within the PoPD/CS network. The characteristic absorption features of PoPD, particularly the visible-region band, often exhibit a wavelength shift (bathochromic (red)/ hypsochromic (blue)) upon composite formation. Such spectral shifts reflect alterations in the electronic environment or variations in the degree of π-conjugation within the PoPD backbone, arising from intermolecular interactions such as hydrogen bonding with the O–H and N–H functionalities of the chitosan matrix.^27^ The spectral observations collectively indicate strong interfacial interactions and effective electronic coupling between PoPD and the CS phase. The optical bandgap (*E*_g_) of the PoPD/CS composite films was determined from the UV–vis absorption spectra using the Tauc plot (given in Supporting Information as Figure S3). Indirect transitions are commonly reported for CS-based polymer systems and conjugated polymer composites due to their predominantly amorphous nature and the presence of localized electronic states near the band edges.^28,29^ The (*ah*ν)^1/2^ plots for the PoPD/CS composite films exhibited a more extended linear region near the absorption edge. The estimated indirect optical bandgap decreased from 2.11 to 1.94 eV as the PoPD content increased from 0.15 to 1 wt %, indicating lower optical bandgap values due to delocalization of electrons over the PoPD backbone. The obtained *E*_g_ values are comparable to those reported for POPD, suggesting homogeneous dispersion and effective interaction of PoPD with the CS matrix.^30^

**Figure 2:**
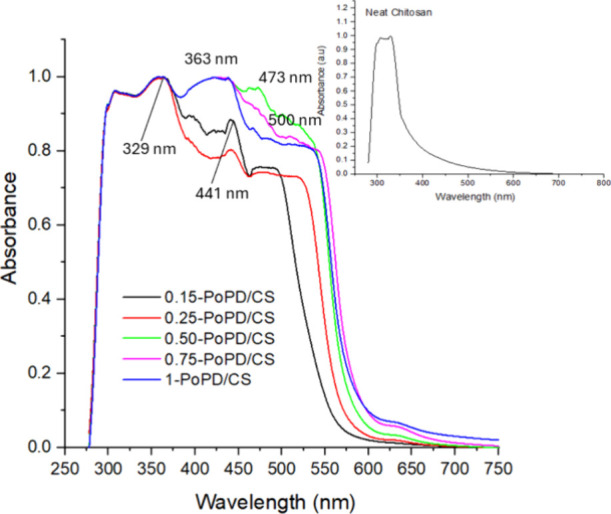
UV–visible spectra of the PoPD/CS composite films.

### 4.3 Fluorescence and Confocal Microscopic Studies

The confocal mapping images of the PoPD/CS composite films (Figure 3a–e) reveal the progressive incorporation and distribution of PoPD nanoparticles within the CS as the PoPD content increases. At lower loadings (a–c), the PoPD particles appear well dispersed, forming a uniform distribution with minimal aggregation, which suggests strong interfacial compatibility and effective stabilization by chitosan. As the PoPD concentration increases (d, e), more distinct and densely packed regions emerge, indicating localized clustering of PoPD domains. These aggregates likely form due to increased particle–particle interactions exceeding the steric and electrostatic stabilization provided by the CS chains. The results suggest that moderate PoPD content enables homogeneous dispersion, while excessive loading promotes phase separation in the composite films. All PoPD/CS composite films display a broad fluorescence emission band centered near 730 nm, corresponding to the deep red to near-infrared region, as seen in confocal imaging (Figure 3f upon excitation at 400 nm. The broad nature of the peak indicates that fluorescence arises from a range of emissive states, a behavior typical of conjugated polymer systems with varying degrees of delocalization. A systematic increase in fluorescence intensity is observed with increasing PoPD loading—from 0.15-PoPD/CS to 1-PoPD/CS composite films, demonstrating a direct correlation between polymer content and emission strength.

**Figure 3:**
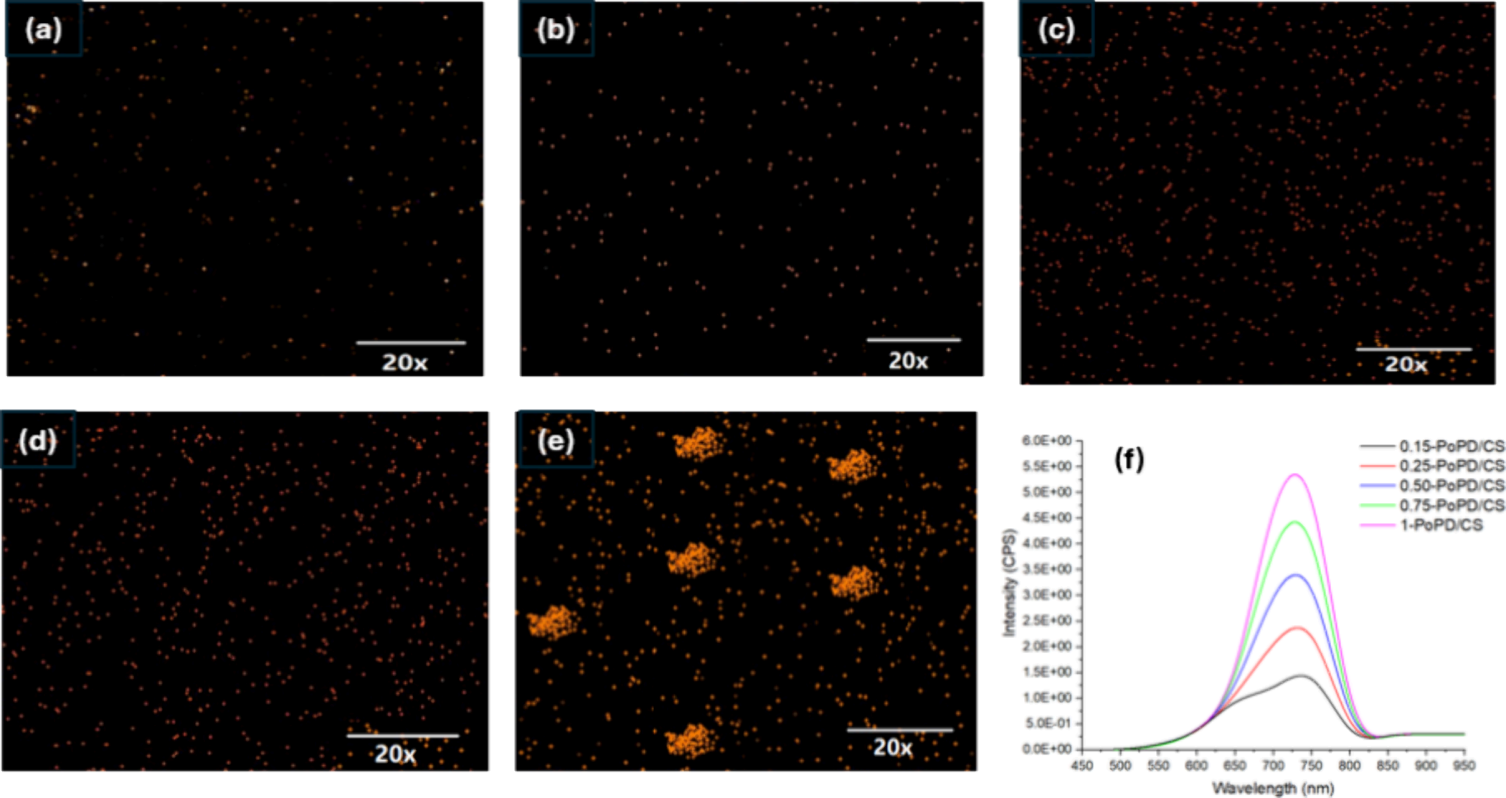
Confocal images of (a) 0.15-PoPD/CS, (b)­0.25-PoPD/CS, (c) 0.50-PoPD/CS, (d) 0.75-PoPD/CS, (e) 1-PoPD/CS, and (f) fluorescence spectra of PoPD/CS composite films.

### 4.4 Morphological Analysis via SEM Studies

SEM images reveal the surface morphology of PoPD/CS composite films with different filler concentrations, as shown in Figure 4a–f. The SEM image of neat CS shown in Figure 4a reveals a dense, relatively uniform, and rough surface morphology. The morphology of the 0.15-PoPD/CS composite film shown in Figure 4b reveals a noticeably network-like surface morphology compared to neat CS. The composite exhibits a more heterogeneous texture characterized by numerous small, rounded features dispersed across the surface. These features likely correspond to the presence of PoPD as a network within the CS matrix. The presence of these dispersed domains suggests successful incorporation of PoPD into the CS matrix but without significant agglomeration at this low concentration. The surface appears rougher and less uniform than neat CS, reflecting the interaction between the biopolymer and the organic filler. This morphology implies an increased interfacial area, which could contribute to altered mechanical and functional properties in the composite material.

**Figure 4:**
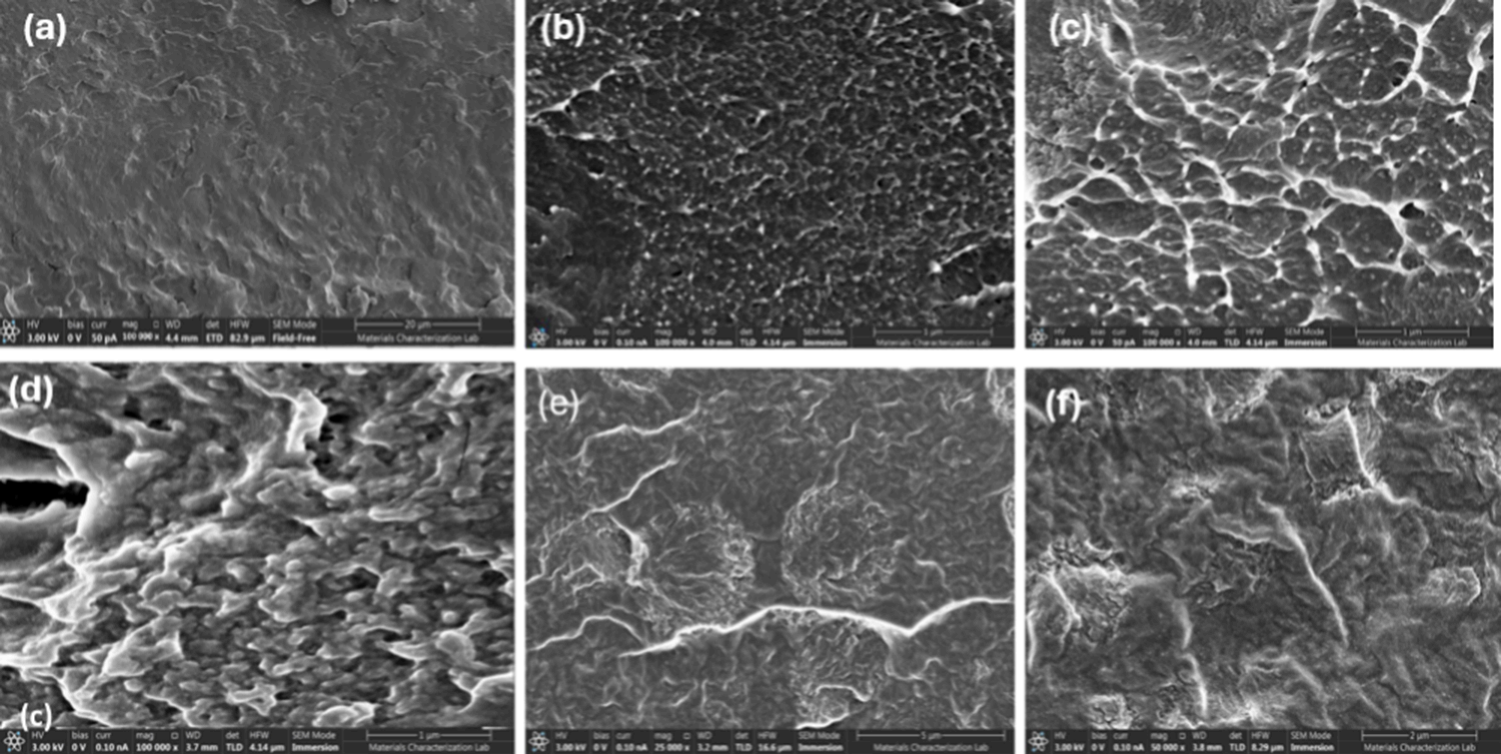
SEM of (a) neat CS, (b) 0.15-PoPD/CS, (c) 0.25-PoPD/CS, (d) 0.50-PoPD/CS, (e) 0.75-PoPD/CS, and (f) 1-PoPD/CS.

The SEM image of the 0.25-PoPD/CS composite films shown in Figure 4c exhibited a highly interconnected, rough network characterized by a nanoporous architecture. This distinct morphology confirmed the effective incorporation of PoPD within the CS matrix. The surface presented web-like structures with well-defined pores, indicating that the polymerization of OPD on the CS framework results in a heterogeneous, interpenetrating polymer network. Additionally, the presence of small aggregated particles or globules, significantly smaller than the main network features, corresponds to PoPD nanoparticles or nanospheres uniformly dispersed throughout the CS matrix. The morphology of the 0.50-PoPD/CS composite film (Figure 4d) also revealed a globular and highly interconnected network structure of PoPD nanospheres that were uniformly dispersed and embedded throughout the CS, creating a composite film with a high surface area. Interestingly, the morphology of the 0.75-PoPD/CS and 1-PoPD/CS composite films (Figure 4e,f) exhibited larger, clearly defined flower-like/spherical self-assembled structures of the PoPD formed as the concentration increased. The cracks visible on the surface surrounding these larger features reflected diminished interfacial adhesion between the polymer aggregates and the CS matrix at very high loadings, leading to a less continuous film compared to that of the lower-concentration composites. The SEM images clearly demonstrated the morphological transition from a smooth biopolymer surface to a textured hybrid composite, with the extent of surface roughness and particle aggregation increasing proportionally to the PoPD content. The incorporation of the filler resulted in a progressively rougher and more interconnected surface morphology, which enhanced mechanical performance at moderate loadings due to improved stress distribution and stronger interfacial bonding. However, at higher loadings, the formation of large aggregates, voids, and microscopic cracks disrupted the structural uniformity of the films. These defects explain the observed behavior in which the elastic modulus partially recovers—owing to the presence of rigid, densely packed domains—while ductility drops significantly because the aggregated structures hinder polymer chain mobility and act as stress-concentration sites.

### 4.5 DMA Studies

DMA analysis was used to obtain a full picture of the dynamic mechanical behavior of CS films and CS films loaded with PoPD at various concentrations (Figure 5a–f). The stress–strain curves of the PoPD/CS composite films illustrate how the incorporation of PoPD influences the mechanical response of the CS matrix. Each curve in Figure 5a–f follows a typical polymeric deformation pattern, beginning with a linear elastic region where stress increases proportionally with strain. This initial segment reflects the material’s stiffness and resistance to deformation. As the strain increases, the curves deviate from linearity, marking the onset of plastic deformation, which arises from molecular chain mobility and interfacial slippage between the PoPD and CS phases.

**Figure 5:**
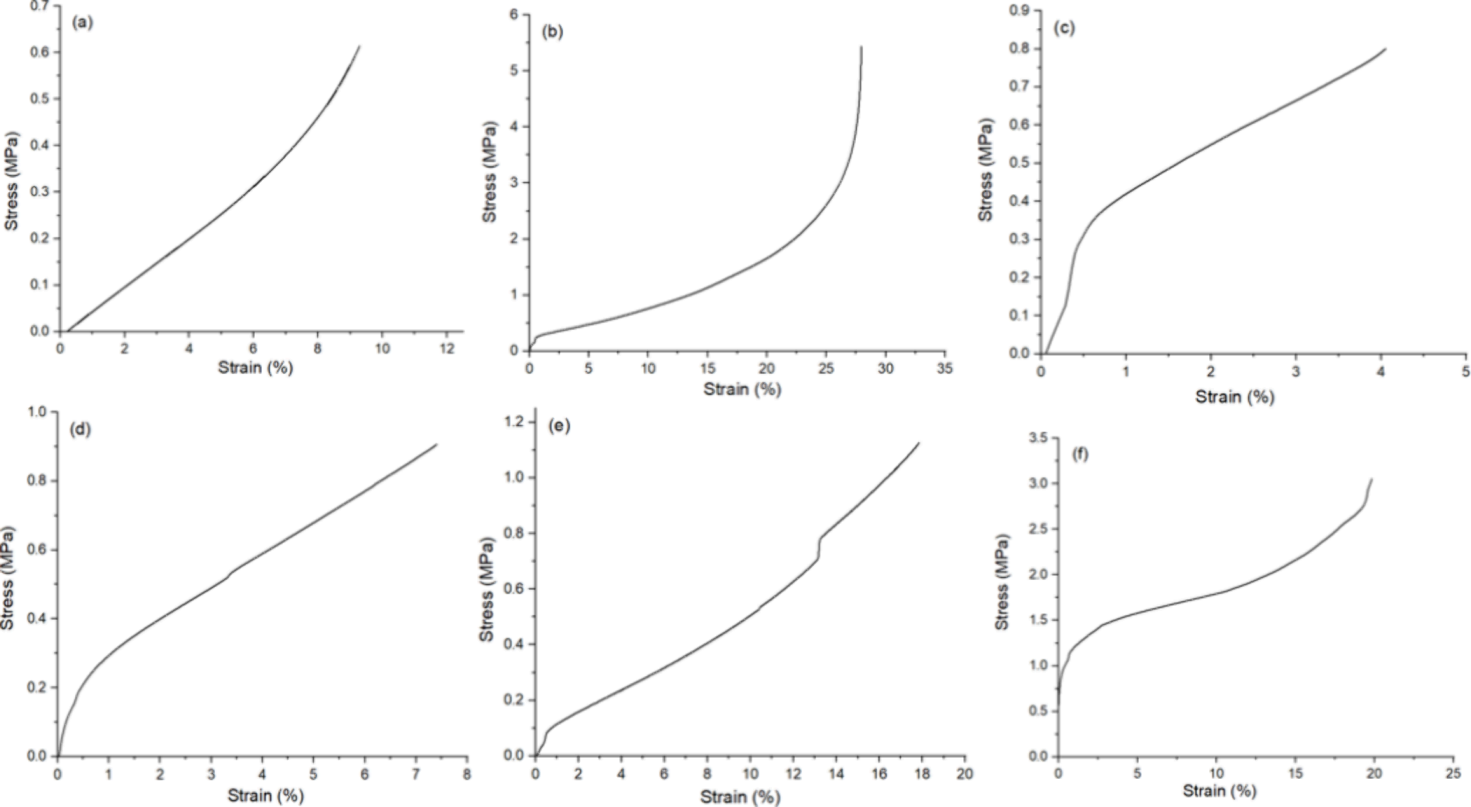
Stress/strain curve of PoPD/CS composite films obtained according to ASTM D882. (a) CS film, (b) 0.15-PoPD-CS, (c) 0.25-PoPD-CS, (d) 0.50-PoPD-CS, (e) 0.75-PoPD-CS, and (f)­1-PoPD-CS.

The composites shown in Figure 5a,c,d show relatively lower stress and limited strain at break, suggesting that these compositions have weaker interfacial interactions, leading to reduced reinforcement efficiency. In contrast, the curves in Figure 5b,e,f display higher tensile strength and greater elongation at break, indicating improved toughness and flexibility. This enhancement can be attributed to stronger hydrogen bonding and π–π stacking interactions between PoPD and CS, which facilitate effective stress transfer during deformation. It is anticipated that strong hydrogen bonding between chain creates a rigid, dense structure, restricting chain movement and contributing to brittleness and fragility; this can be characterized by low elongation at break and moderate tensile strength.^31^ Neat CS exhibited a tensile strength of ∼ 10 MPa, which aligns with literature reports,^32^ as shown in Figure 6a. Higher values but lower tensile strength (∼30–60 MPa) have been associated with homogenization, crystalline alignment, and structural reinforcement strategies.^25^ Using PoPD as a reinforcement filler follows the trend seen in CS composites, where low polymer loading enhances performance, as shown in Figure 6b. Neat CS films exhibited brittle behavior, fracturing at a very low elongation at break of 0.61 ± 0.03% and displaying a comparatively low tensile strength of 9.28 ± 0.46 MPa. The incorporation of PoPD led to a pronounced enhancement in mechanical performance. At 0.15 wt % PoPD, the films showed a substantial increase in ductility and strength, with an elongation at break of 5.44 ± 0.27%, a tensile strength of 27.98 ± 1.40 MPa, and a Young’s modulus of 1840 ± 92 MPa (Figure 6c). In contrast, films containing higher PoPD loadings (0.75- and 1-PoPD/CS) exhibited reduced tensile strength (17.86 and 19.82 MPa, respectively) and lower elongation at break (1.13 and 3.05%, respectively), while the Young’s modulus increased to approximately 2396–2301 MPa (Figure 6c), indicating a transition toward stiffer but more brittle behavior.

**Figure 6:**
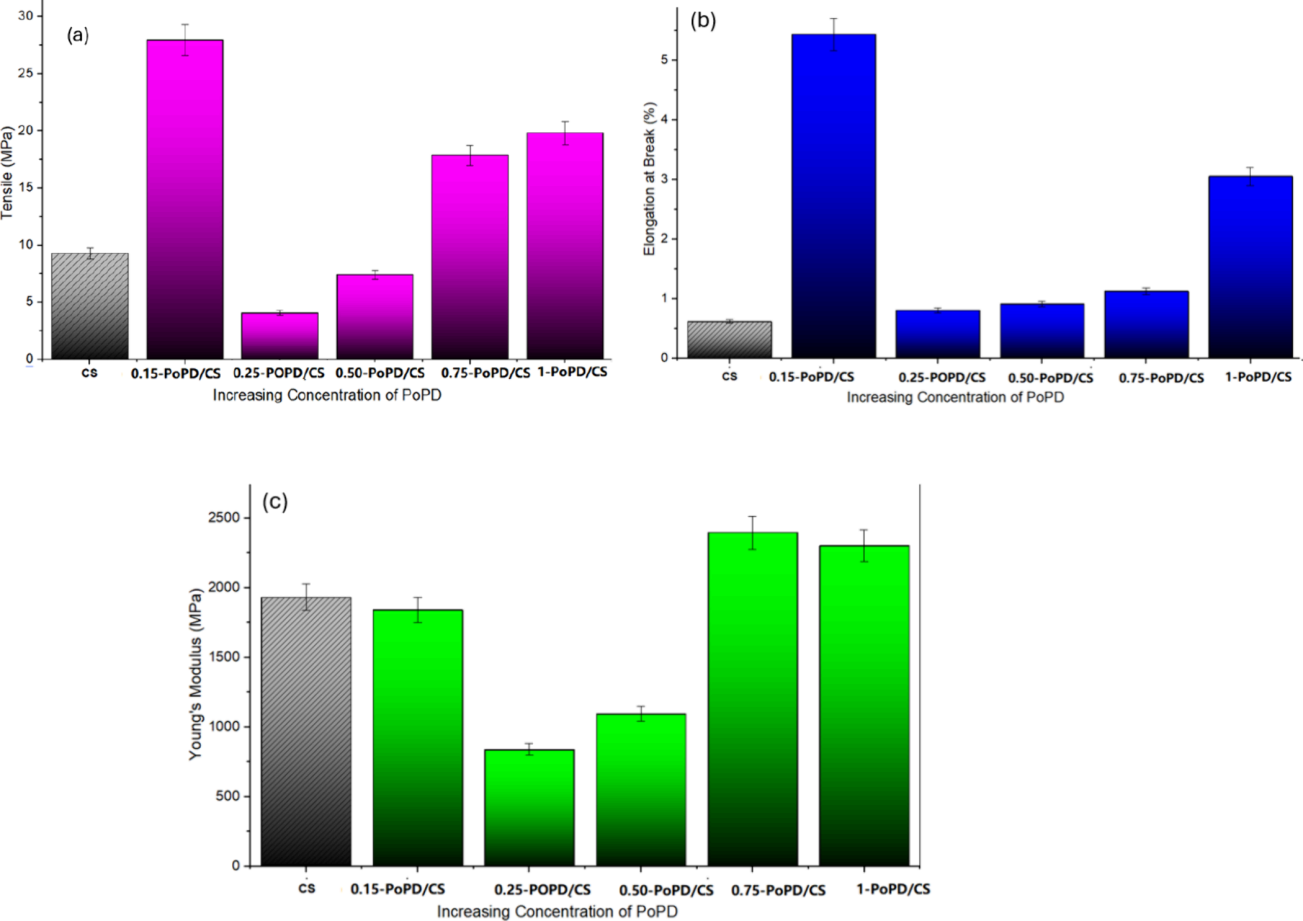
(a) Tensile strength vs PoPD concentration. (b) Elongation at break vs PoPD concentration. (c) Young’s modulus vs PoPD concentration.

From the comparative data shown in Table 1, it can be observed that the PoPD/CS composite films in the present study operate in a much lower organic filler concentration regime. At 0.15 wt % PoPD, the composite exhibits a balanced combination of tensile strength (∼28 MPa), moderate elongation (∼5.4%), and Young’s modulus (∼1840 MPa), comparable to several higher-loading PANI/CS systems. Compared to conventional PANI/CS composites that rely on relatively high PANI contents to achieve reinforcement, the PoPD/CS composite films demonstrate the low-concentration organic fillers that impart appreciable mechanical enhancement while minimizing agglomeration and excessive loss of ductility. This highlights the advantage of PoPD as an efficient reinforcing filler and its influence on the overall structure–property relationship of the composite at low filler loadings.

**Table 1:** Comparative Table of CS Composite Films

CS film composition	PANI loading/concentration	tensile strength (MPa)	elongation at break (%)	Young’s modulus (MPa)
PANI/CS nanocomposite^15^	0.1 M aniline	40.5	28.0	1950
PANI/CS nanocomposite^15^	0.3 M aniline	52.7	23.0	2310
PANI/CS nanocomposite^15^	0.5 M aniline	62.5	18.0	2750
PANI/CS blend (undoped)^16^	emeraldine base (EB)	56.8	6.2	2150
PANI/CS blend (doped)^16^	1.0 M HCl doping	∼39.0	∼4.3	∼1505
ternary CS–PANI-PVA^17^	75:25 (CS:PANI ratio)	40.0–45.0 + 1	50.0–120.0	—
0.15-POPD/CS	0.15 wt %	27.98 ± 1.40	5.44 ± 0.27	1840 ± 92
0.75-POPD/CS	0.75 wt %	17.86	1.13	2396

### 4.6 Moisture Absorption and Radical Scavenging Studies

Figure 7a presents the time-dependent moisture absorption behavior of chitosan (CS) and PoPD/CS composite films. The CS film exhibited a continuous increase in water update from 6.9% at 1 h to 17.2% at 24 h, followed by a decrease to 12.1% at 48 h, suggesting the attainment of a saturation state and partial structural relaxation. In contrast, the incorporation of PoPD altered the moisture absorption behavior, displaying earlier saturation with a lower equilibrium moisture uptake. The 0.15-PoPD/CS composite film reached a stable absorption level of ∼7.7% by 4 h and remained unchanged up to 48 h, while the 0.25-PoPD/CS composite film showed a consistent uptake of ∼10% from 2 h onward. The 0.50-PoPD-CS film exhibited the highest initial uptake (18.2% between 2 and 24 h) before decreasing to ∼9% at 48 h, which is likely attributed to structural heterogeneity that facilitated water penetration. The 0.75-PoPD/CS composite film remained stable at ∼9% throughout the study period, whereas the 1 wt % PoPD-CS film exhibited minimal moisture absorption at 1–2 h with moderate update (6.7%) at 4–6 h.

**Figure 7:**
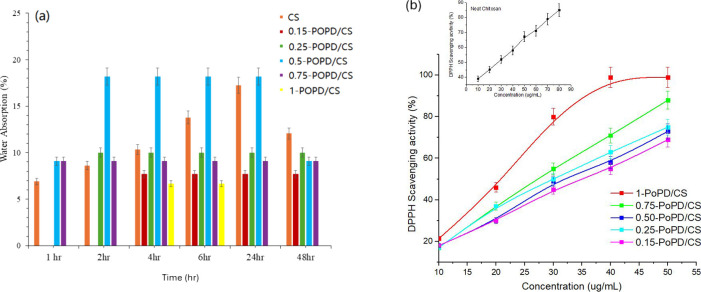
(a) Moisture absorption plot of PoPD/CS. (b) % DPPH activity of PoPD/Cs composite films.

Overall, the reduced moisture uptake of PoPD/CS composite films compared to neat CS demonstrates that PoPD incorporation improves moisture resistance, particularly at low loadings. This behavior is attributed to the formation of additional intermolecular hydrogen bonds, which promote moisture saturation by limiting accessible hydrophilic sites. This trend mirrors the balance observed in the mechanical and SEM analyses: even 0.15 wt % PoPD improves structural integrity without disrupting transparency, while excessive loading produces aggregated, dense morphologies that alter barrier behavior. The antioxidant activities of the composites were determined by evaluating their ability to scavenge the DPPH radical based on the decrease in absorbance at 516–517 nm, as shown in Figure 7(b). The scavenging ability was concentration-dependent. For CS, the scavenging activity increased from 40.4 to 80.6%, with an increase in concentration from 10 to 80 μg/mL. Among the PoPD/CS composite films, the highest scavenging activity was noticed for 1-PoPD/CS, which was ∼90% at 50 μg/mL, indicating that the composite films exhibited stronger scavenging activity for DPPH radicals than CS alone.

### 4.7 Antimicrobial Studies via Disc Diffusion

The antibacterial efficacy of PoPD/CS composite films was evaluated using the disc diffusion method against *B. subtilis*, a Gram-positive model organism commonly used in food-packaging-related studies.^33,34^ The thick peptidoglycan layer of *B. subtilis*, enriched with negatively charged teichoic acids, provides a relevant target for the polycationic antimicrobial mechanism of CS-based materials.^35^ As shown in Figure 8(a), PoPD-CS films inhibited the growth of *B. subtilis* in a concentration-dependent manner, with all composite films exhibiting larger inhibition zones (cm) than pristine CS (Figure 8a,b). One-way ANOVA confirmed a significant overall difference between the groups (*F* = 14.13, *p* = 4.08 × 10^–8^). A subsequent pairwise *t-*test revealed that the inhibition zones were significantly greater than the CS control for all PoPD-CS composites tested. Specifically, the 0.15-PoPD/CS, 0.25-PoPD/CS, 0.50-PoPD/CS, and 1-PoPD/CS films showed statistically significant increases compared to CS, while the 0.75-PoPD/CS films showed a moderate increase (*p* < 0.01). The greatest inhibition was observed for 0.15-PoPD/CS (1.68 ± 0.13 cm), proposing that lower PoPD loading within the CS matrix may optimize antibacterial performance.

**Figure 8:**
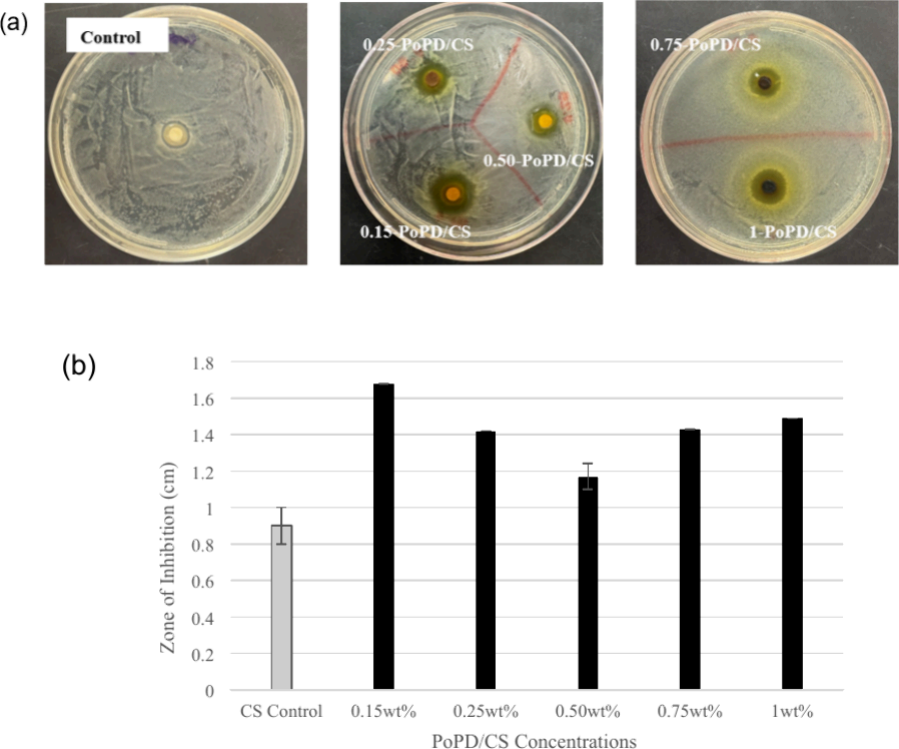
(a) Disc diffusion zones for CS and PoPD/CS. (b) Zone of inhibitions of PoPD-CS films against *B. subtilis*. Data are the mean ± SD (*n* = 9 for PoPD-CS, *n* = 3 for CS). All concentrations were significantly higher than CS (*p* < 0.01), with the greatest activity at 0.15- PoPD/CS (*p < 0.001 vs CS).

### 4.8 Docking Studies

Docking studies showed that both the OPD monomer (Figure 9a) and the PoPD/CS composite fibers (Figure 9b,c) can attach to the DNA gyrase A protein in *B. subtilis* (PDB: 4DDQ). This molecular docking approach helps qualitatively analyze how PoPD-CS interacts with bacterial proteins. The use of docking to access binding to key enzymatic targets is increasingly applied to CS-based materials.^36,37^ The abundance of hydroxyl and amino groups in CS facilitates stabilization via hydrogen bonding and electrostatic interaction. These behaviors are well-documented in CS conjugates with enhanced antimicrobial action.^38^

**Figure 9:**
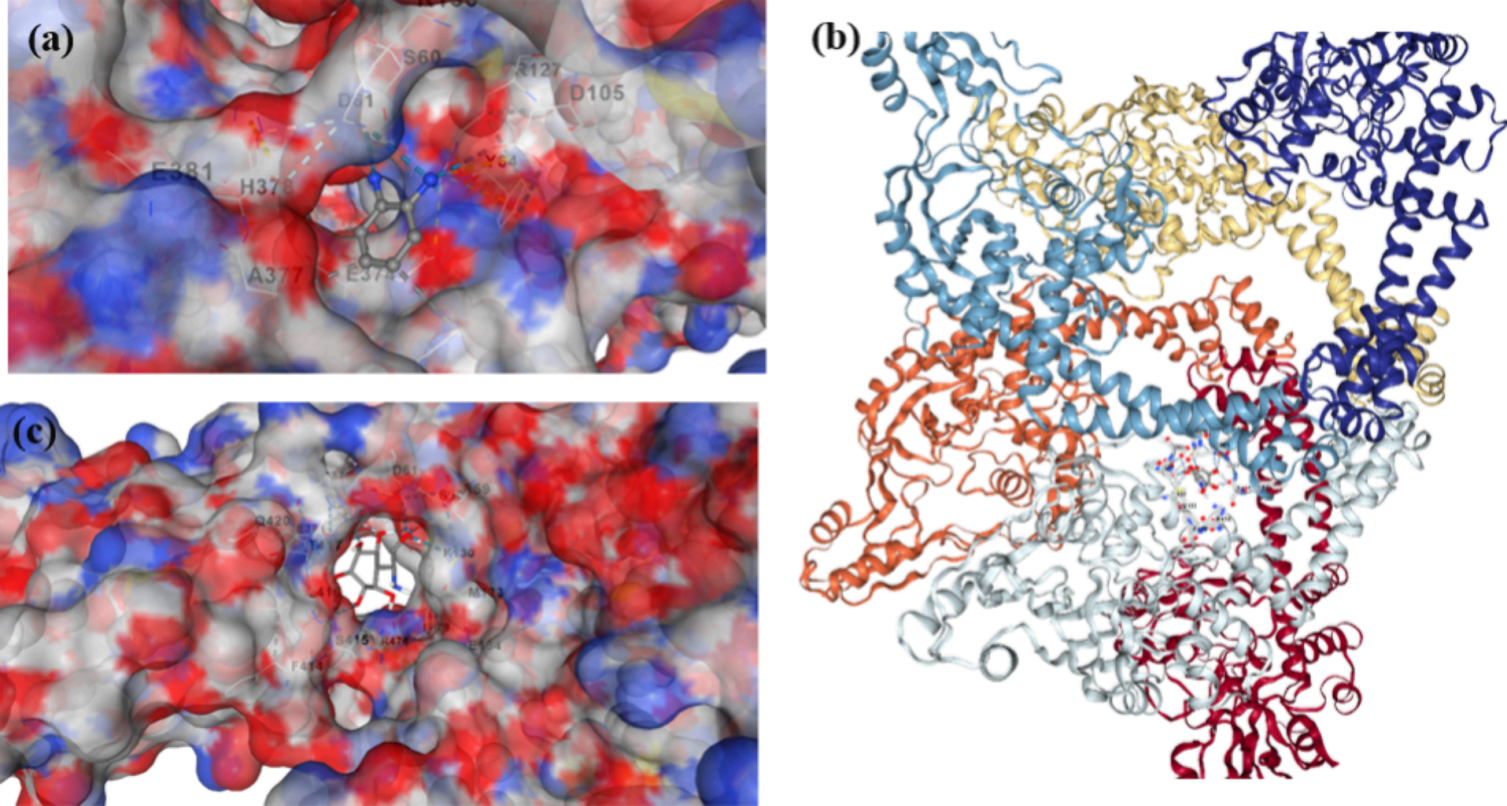
3D images of docking of *B. subtilis* with the OPD monomer (a), *B. subtilis* with PoPD/CS shown in helices form (b) image of PoPD/CS within cavity C1 of *B. subtilis* (c).

The docking analysis identified five possible surface cavities with calculated binding energies in the range of −4.2 to −4.5 kcal·mol^–1^, which are indicative of weak to moderate, nonspecific interactions rather than strong target-specific binding (table provided in Supporting Information as table S2). Among these sites, Cavity 1 exhibited the largest accessible volume (≈3578 Å^3^) and the most favorable docking score (−4.5 kcal·mol^–1^), suggesting that it may accommodate polymeric segments more readily due to geometric compatibility rather than high-affinity molecular recognition. The protein surface within this cavity contains a heterogeneous distribution of charged, polar, and hydrophobic residues, including negatively charged residues (e.g., GLU381, ASP61), positively charged residues (e.g., LYS455, ARG127), and neutral or aromatic residues. This chemical diversity creates a general interaction landscape that can support transient electrostatic, hydrogen-bonding, and π–π interactions with the PoPD–CS composite. In particular, the aromatic backbone of PoPD may engage in weak π–π interactions with aromatic residues such as HIS378 and PHE414, while hydroxyl and amino groups from the chitosan matrix may participate in hydrogen bonding with polar residues (e.g., SER415). Electrostatic complementarity between protonated amine groups in CS/PoPD and oppositely charged amino acid residues may further contribute to surface association. Importantly, these interactions should be interpreted as nonspecific surface associations rather than evidence of selective binding to a defined biological target. Given the relatively low docking energies, the results do not support a dominant inhibition mechanism involving a single intracellular protein. Instead, the docking analysis is presented here only as qualitative, supportive evidence, suggesting that PoPD–CS composites can interact with biomolecular surfaces. Accordingly, the experimentally observed antibacterial activity is more appropriately attributed to well-established mechanisms for CS- and conducting-polymer-based materials including electrostatic interactions with bacterial membranes, disruption of membrane integrity, increased permeability, and contact-mediated antimicrobial effects. The docking results complement these observations by indicating that weak, nonspecific biomolecular interactions may occur upon contact rather than defining a specific molecular target.

## 5 Conclusions

PoPD/CS composite films were successfully synthesized via in situ polymerization and evaluated for their structural, mechanical, optical, and functional performance. Spectroscopic and morphological analyses confirmed the addition of POPD as a filler in the CS matrix, with morphological evolution from porous structures in CS to smooth and heterogeneous surfaces at higher PoPD loadings. Neat CS films exhibited limited ductility and modest tensile strength (9.28 ± 0.46 MPa, elongation at break 0.61 ± 0.03%), whereas the incorporation of 0.15 wt % PoPD resulted in a substantial enhancement in tensile strength (27.98 ± 1.40 MPa) and elongation at break (5.44 ± 0.27). Higher PoPD loadings increased Young’s modulus up to ∼2400 MPa but revealed less consistency in ductility presumably to aggregation of the POPD filler in the CS matrix, as seen in the SEM studies. Moisture uptake in CS was reduced from ∼17–20% for CS to ∼7–10% for PoPD-CS films. Antibacterial assays against *B. subtilis* confirmed statistically significant inhibition for all PoPD-CS composite films (*p* < 0.01), with optimal activity observed at 0.15 wt % (inhibition zone of 1.68 ± 0.13 cm). In parallel, antioxidant performance with DPPH scavenging was also enhanced, increasing from ∼80.6% for CS to ∼90% for 1-PoPD-CS at 50 μg/mL. Overall, the mechanical, surface, and functional evaluations demonstrate that incorporating PoPD at levels as low as 0.115 wt % provides good improvements in tensile strength, flexibility, moisture stability, antioxidant performance, and antibacterial activity. These results highlight PoPD/CS composite films as versatile multifunctional materials with strong potential for use in biocompatible coatings and smart food-packaging applications.

## Supplementary Material



## Notes

Calibration curve and UV–visible spectrum of ascorbic acid (Figure S1); FTIR spectra of CS and PoPD/CS films (Figure S2); Tauc plots of PoPD/CS films with varying PoPD loadings (Figure S3); colorimetric analysis of PoPD/CS films (Table S1); and molecular docking cavity and interaction details of protein 4DDQ with PoPD/CS films (Table S2) (PDF)
