# Supplementary material for: Poly(o‑phenylenediamine) as an Organic Filler for Enhancing the Mechanical and Antibacterial Performance of Chitosan Films
Source: ACS Appl Bio Mater. 2026 Feb 5;9(4):2250–62. doi: 10.1021/acsabm.5c02312 (PMC12914638; doi:10.1021/acsabm.5c02312)
Supplement: Supplementary file 1 [file mt5c02312_si_001.pdf]

Supporting Information

Poly(o-phenylenediamine) as an Organic Filler for Enhancing the Mechanical and Antibacterial Performance of Chitosan Films

Mary Taylor [1,2], Jayla Jenkins [1], Mohammad Mohiuddin [3] and Ufana Riaz [1,2]*
[1] Biomedical/Biotechnology Research Institute, North Carolina Central University, Durham, NC, USA
*Corresponding author: uriaz@nccu.edu
[2] Julius L. Chambers Biomedical/Biotechnology Research Institute (BBRI), North Carolina Central University, 1801 Fayetteville St. Durham, NC 27707
[3] School of Packaging. Michigan State University 110 Packaging Building 448 Wilson Road East Lansing, MI 48824-1223

Title of Table/Figure	Pg no
Figure S1 (a) Calibration plot of ascorbic acid (b) UV-visible plot of ascorbic acid used as control for DPPH scavenging activity.	S2
Figure S2 Fourier transform infrared (FT-IR) spectra of CS and PoPD/CS films.	S3
Figure S3 Tauc plots of (a) 0.15-PoPD/CS, (b) 0.25 -PoPD/CS, (c) 0.5-PoPD/CS, 0.75-PoPD/CS, 1-PoPD/CS.	S4
Table S1 Colorimetric analysis of PoPD CS films	S5
Table S2 Summary of the docked cavity of carbons (1-5) and contact sites of protein 4DDQ with PoPD/CS films	S5

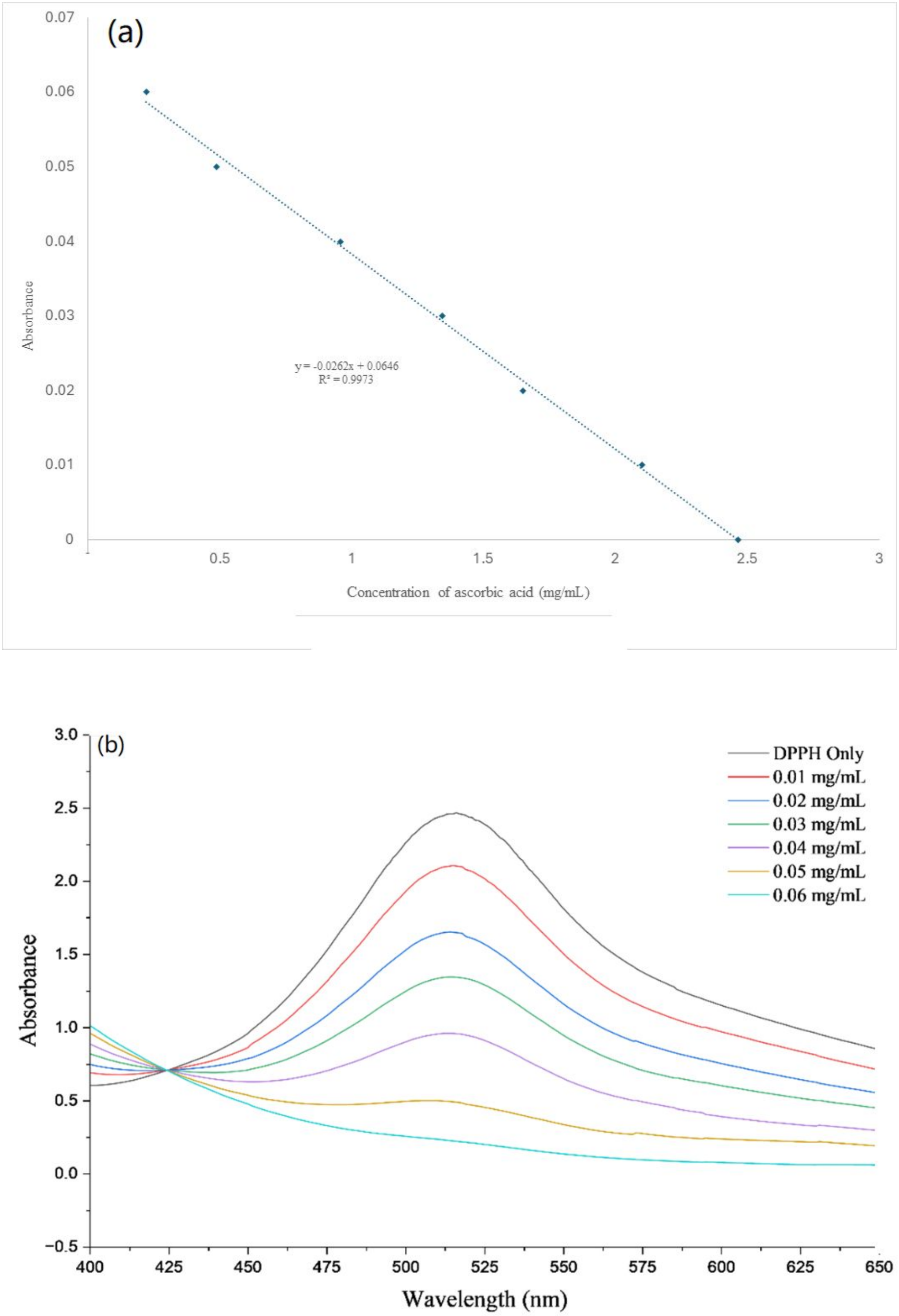

Figure S1 (a) Calibration plot of ascorbic acid (b) UV-visible plot of ascorbic acid used as control for DPPH scavenging activity.

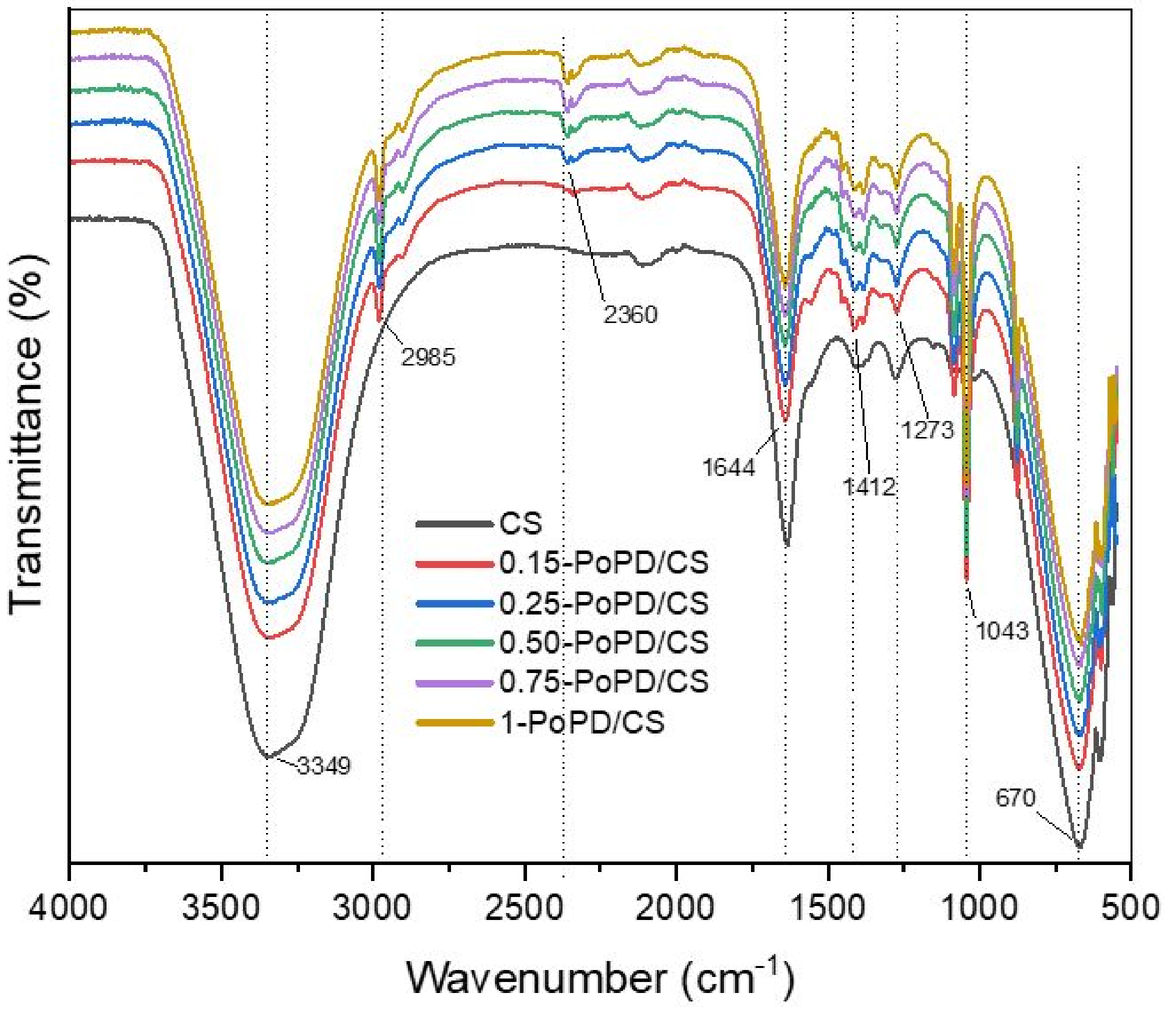

Figure S2 Fourier transform infrared (FT-IR) spectra of CS and PoPD/CS films.

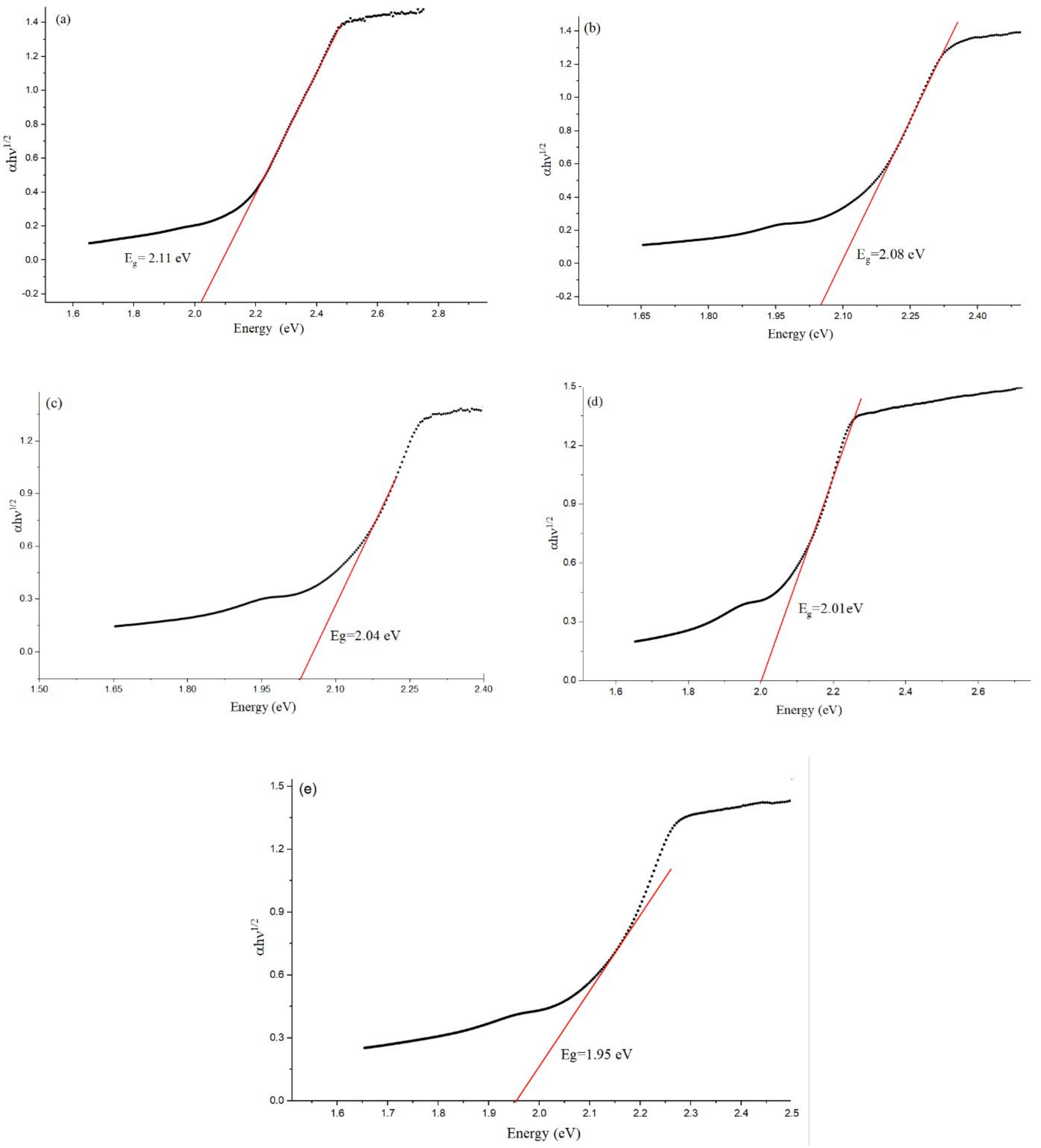

Figure S3 Tauc plots of (a) 0.15-PoPD/CS, (b) 0.25 -PoPD/CS, (c) 0.5-PoPD/CS, 0.75-PoPD/CS, 1-PoPD/CS.

Table S1 Colorimetric analysis of PoPD CS films

Sample	Thickness (mm)	L∗ (Lightness)	a∗ (Red-Green)	b∗ (Yellow-Blue)	ΔE (vs Film CS)	ΔE (vs Previous)
CS	0.0124	82.55	5.7	69.90	—	—
0.15-PoPD/CS	0.0127	70.65	30.58	65.77	27.87	27.87
0.25-PoPD/CS	0.0125	67.20	38.37	57.02	38.31	12.22
0.5-PoPD/CS	0.0134	63.70	41.63	48.86	45.69	9.46
0.75-PoPD/CS	0.0136	64.56	41.79	51.32	44.39	2.61
1-PoPD/CS	0.0139	58.55	22.74	27.36	51.73	31.19

Table S2 Summary of the docked cavity of carbons (1-5) and contact sites of protein 4DDQ with PoPD/CS films

ID	Volume (Å³)	Center Coordinates (x, y, z)	Vina Score	Highlighted Residues
**1**	3578	(-1.61, -62.51, -20.26)	-4.5	GLU:374:B, ALA:375:B, ALA:377:B, HIS:378:B, GLU:381:B, GLY:382:B, VAL:385:B, PHE:414:B, SER:415:B, LEU:416:B, THR:417:B, GLN:420:B, VAL:448:B, ILE:451:B, LYS:455:B, THR:59:C, SER:60:C, ASP:61:C, TYR:64:C, ASP:105:C, ARG:127:C, LYS:130:C, MET:133:C, GLU:134:C, ARG:478:C
**2**	3235	(-48.49, -42.45, -40.51)	-4.4	SER:60:D, ASP:61:D, SER:129:D, LYS:130:D, ILE:131:D, MET:133:D, GLU:134:D, ARG:137:D, ARG:365:D, GLU:470:D, GLU:471:D, GLU:474:D, ARG:478:D, PHE:479:D, HIS:378:E, GLU:381:E, GLY:382:E, VAL:385:E, ILE:411:E, GLU:412:E, GLN:413:E, PHE:414:E, SER:415:E, LEU:416:E, THR:417:E, GLN:420:E
**3**	2283	(8.31, -75.70, -43.72)	-4.4	ASP:37:C, VAL:38:C, ARG:39:C, ARG:47:C, GLN:147:C, VAL:158:C, VAL:159:C, LEU:337:C, VAL:338:C, ASP:339:C, GLY:340:C, GLN:341:C, LYS:343:C, HIS:353:C, TYR:354:C, ASP:356:C, HIS:357:C, VAL:360:C, LYS:66:F, GLY:106:F, HIS:107:F, GLY:108:F, ASN:109:F, PHE:110:F, GLY:111:F, SER:112:F, ASP:116:F, ARG:122:F, TYR:123:F, THR:124:F, GLU:125:F, ARG:298:F
**4**	1567	(-59.73, -42.25, -13.13)	-4.2	TYR:64:A, LYS:65:A, LYS:66:A, ARG:69:A, HIS:107:A, GLU:125:A, LYS:66:B, ARG:122:B, TYR:123:B, SER:32:D, ARG:33:D, ALA:34:D, ASP:37:D, ARG:39:D, LYS:43:D, PRO:44:D, ARG:47:D, ARG:48:D, PRO:80:D, HIS:81:D, ARG:92:D, GLN:147:D, ASP:148:D, ASN:149:D, TYR:150:D, ASP:151:D, GLY:152:D, GLU:156:D, PRO:157:D,

				VAL:158:D, VAL:159:D, ALA:173:D, GLY:174:D, ILE:175:D, LEU:337:D
**5**	1093	(-58.14, -31.09, -16.68)	-4.3	ALA:94:A, GLN:95:A, ASP:96:A, PHE:97:A, GLY:106:A, HIS:107:A, GLY:108:A, ASN:109:A, PHE:110:A, GLY:111:A, SER:112:A, ALA:119:A, TYR:123:A, THR:124:A, ALA:34:D, LEU:35:D, PRO:36:D, ASP:37:D, ARG:39:D, LEU:42:D, LYS:43:D, PRO:44:D, ARG:47:D, ARG:48:D, GLN:147:D, GLU:156:D, PRO:157:D, VAL:158:D, VAL:159:D, LEU:337:D, ASP:339:D, GLY:340:D, GLN:341:D
